# The EXIT for Prenatally Diagnosed Cervical Cystic Teratoma: A Case Report

**Published:** 2015-04-01

**Authors:** Sabri Cansaran, Ayşenur Cerrah Celayir, Serdar Moralıoğlu, Habibe Ayvacı, Semih Tuğrul, Fahri Ovalı, Handan Çetiner

**Affiliations:** 1 Department of Paediatric Surgery, Zeynep Kamil Maternity and Children’s Training and Research Hospital, Istanbul; 2 Department of Perinatology, Zeynep Kamil Maternity and Children’s Training and Research Hospital, Istanbul; 3 Department of Neonatology, Zeynep Kamil Maternity and Children’s Training and Research Hospital, Istanbul; 4 Department of Pathology, Zeynep Kamil Maternity and Children’s Training and Research Hospital, Istanbul

**Keywords:** EXIT, Cervical teratoma, Prenatal diagnosis, Prenatal MRI

## Abstract

The Ex-utero intrapartum treatment (EXIT) is a procedure performed during caesarean section while on fetal-placental circulation. We present a prenatally diagnosed cervical cystic mass causing tracheal compression which was managed successfully with the EXIT procedure.

## CASE REPORT

A 26-year-old pregnant was referred to our hospital due to polyhydramnios and fetal cervical cystic mass. Council of Perinatology decided to organize a fetal MRI, an elective caesarean section with EXIT procedure. On fetal MRI, 6x8x10 cm cervico-mandibular mass was noted; it contained both solid and cystic components and it pushed the trachea to the right side. It was reported as cystic lymphangioma or teratoma by fetal MRI (Fig.1). 

**Figure:**
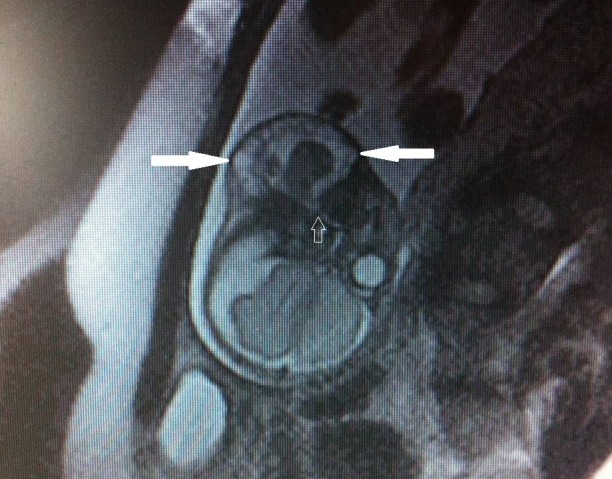
Figure 1: Fetal MRI showing a cervico-mandibular mass.

At 36 weeks of gestation, caesarean section (C/S) and EXIT procedure were carried out with endotracheal intubation of infant with a No.3 endotracheal tube, before clamping of umbilical cord (Fig.2). A 3090 gram male newborn was followed up in surgical NICU with mechanical ventilation. A cystic mass, approximately 10 cm in diameter, was seen in the left side of the upper neck, which displaced left mandible laterally and extended up to the left tragus; a diagnosis of cystic lymphangioma was made. About 100 ml of clear fluid from the cyst was aspirated and 3 mg bleomycin was injected into the cyst. The fluid re-accumulated in the cyst next 24 hours. On 5th day of life, the cystic mass was completely excised (Fig.3) through a submandibular transverse incision; the vessels and nerves of the neck were carefully preserved. The neonate was extubated on the 2nd postoperative day (POD), and drain was removed on 4th POD. The neonate was fed through a nasogastric tube till 7th POD. 

**Figure:**
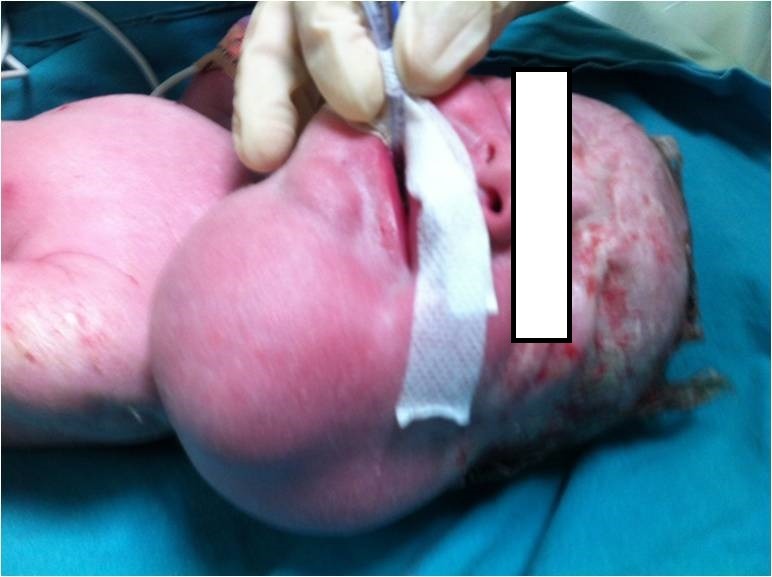
Figure 2: Airway secured during EXIT.

**Figure:**
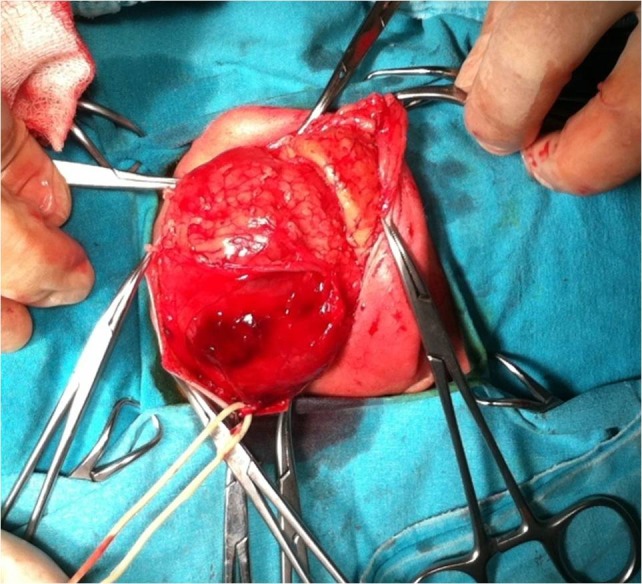
Figure 3: The cystic mass was excised totally with preserving of vessels and nerves of the neck by submandibular transverse incision.

Histopathological diagnosis was mature cystic teratoma. At 6-month follow-up, there was no recurrence, and the mandibular dislocation had significantly decreased. Alpha-fetoprotein levels declined from 34,000 ng/ml to 34 ng/ml over this period.

## DISCUSSION

The EXIT procedure was described first in 1990 [1,2] for the management of newborns diagnosed antenatally with extrinsic or intrinsic obstructive malformations of upper airway. Most of the lesions in cervical area are benign, but they may cause mortality due to airway obstruction. The EXIT is commonly used in such situations for maintaining and securing adequate fetal oxygenation during the time needed for airway handling (laryngoscopy, bronchoscopy, tracheal intubation or tracheotomy).[1,2]

A well-defined, heterogeneous mass on antenatal USG at the anterolateral portion of the fetal neck with hyperextension of the neck is characteristic for the cervical teratoma. [3-6] Prenatal MRI is important for further elaboration of such masses around airway with reference to its extent and compression on the airway. [5,6] Our case was prenatally diagnosed as cervical cystic teratoma or cystic lymphangioma and an EXIT was planned because of expected difficulty of airway maintenance. 

Prenatal diagnosis of cervical masses with upper airway obstruction should alert the perinatal team for closed and coordinated management. Appropriate timing and mode of delivery, safe airway, and timely surgical intervention are crucial for the survival of neonates. Although prenatal diagnosis has not yet affected mortality of cervical teratoma, it is hoped that with further experience and management team approach, mortality can be reduced.[4] The team approach is almost importance in providing organized and coordinated care plan. An obstetrician and/or perinatologist, neonatologist, paediatric surgeon and if necessary, paediatric anaesthesiologist should all be available in the delivery room for the birth, resuscitation and possible surgical intervention to the neonate.[4-6]

A C/S with EXIT procedure is indicated to avoid dystocia and/or teratoma avulsion especially for large tumours. [5] A careful anaesthetic management is necessary to provide oxygenation of fetus during C/S and to maintain a patent airway after delivery. In our case the EXIT was successfully executed and the airway was secured with No.3 endotracheal tube. The elective surgical excision of the mass was performed completely from surrounding tissues. Respiratory distress persisted until the fourth day postoperatively, and the newborn was extubated on the fourth day.

## Footnotes

**Source of Support:** Nil

**Conflict of Interest:** None

